# Determinantes sociales de la salud: propuesta explicativa alternativa al enfoque biomédico de la conducta suicida

**DOI:** 10.15446/rsap.V26n1.116420

**Published:** 2024-01-01

**Authors:** Ladini Sunanda Hernández-Bello, Fernando Pio De La Hoz Restrepo, Zuleima Cogollo-Milanés

**Affiliations:** 1 LH: Enf. M. Sc. Enfermería en Salud Mental. Ph. D. (c). Salud Pública. Docente, Facultad de Enfermería, Universidad de Cartagena. Cartagena, Colombia. ladinihernandez3126@gmail.com Universidad de Cartagena Facultad de Enfermería Universidad de Cartagena Cartagena Colombia; 2 FH: MD. M. Sc. Edipemiología. Ph. D. Epidemiología. Docente, Facultad de Medicina, Departamento de Salud Pública. Universidad Nacional de Colombia. Bogotá, Colombia. fpdelahozr@unal.edu.co Universidad Nacional de Colombia Facultad de Medicina Departamento de Salud Pública Universidad Nacional de Colombia Bogotá Colombia fpdelahozr@unal.edu.co; 3 ZC: Enf. M. Sc. Salud Pública. M. Sc. Enfermería Salud Mental. Ph. D. Salud Pública. Docente, Facultad de Enfermería, Universidad de Cartagena. Cartagena, Colombia. zcogollom@unicartagena.edu.co Universidad de Cartagena Facultad de Enfermería Universidad de Cartagena Cartagena Colombia zcogollom@unicartagena.edu.co

**Keywords:** Suicidio, intento de suicidio, determinantes sociales de la salud, epidemiología social *(fuente: DeCS, BIREME)*, Suicide, suicide attempt, social determinants of health, social epidemiology *(source: MeSH, NLM)*

## Abstract

La conducta suicida representa un grave problema de salud pública, las muertes por suicidio se estiman cada año en 800 000 personas. De ahí que su estudio haya cobrado relevancia, en la medida en que se requiere comprender el fenómeno, sus causas y las posturas teóricas que orientan la toma de decisiones y el diseño de estrategias de prevención. Sin embargo, la explicación hegemónica justifica la ocurrencia del fenómeno desde la interacción de múltiples factores de riesgo, en los cuales se fundamentan diversos modelos explicativos con enfoque biomédico-céntrico, que postulan la conducta suicida como una consecuencia de la psicopatología individual. Este ensayo desarrolla una crítica a la postura teórica hegemónica biomédica y resalta las bondades que tendría el estudio del fenómeno desde una perspectiva social, específicamente desde el modelo de los determinantes sociales de la salud de la Organización Mundial de la Salud, sin desmeritar los aportes teóricos y empíricos del enfoque biomédico, pero sí destacando sus debilidades y resaltando las fortalezas del enfoque social, que sin dejar de lado los factores individuales, ofrece una visión más integral en la comprensión de la conducta suicida.

## Abstract (en)

Suicidal behavior represents a serious public health problem. Suicidal deaths are estimated at 800,000 people each year. This is why its study has gained relevance to the extent that it is necessary to understand the phenomenon, its causes and theoretical positions that guide decision-making and the design of prevention strategies. However, the hegemonized explanation justifies the occurrence of the phenomenon from the interaction of multiple risk factors, on which various explanatory models with a biomedical-centric approach are based, which postulate suicidal behavior as a consequence of individual psychopathology. This essay develops a critique of the hegemonic biomedical theoretical position and highlights the benefits of studying the phenomenon from a social perspective, specifically from the World Health Organization's model of social determinants of health, without undermining the theoretical and empirical contributions of the biomedical approach, but highlighting its weaknesses and emphasizing the strengths of the social approach, which, without leaving aside individual factors, offers a more comprehensive view in understanding suicidal behavior.

Los intentos de suicidio y el suicidio consumado representan un grave problema de salud pública, cada año 800 000 personas se suicidan y es la tercera causa de muerte entre los 15 y los 19 años. Además, se estima que por cada sujeto que muere por suicidio 20 personas lo han intentado. El suicidio en cualquier etapa de la vida, más aún en la juventud, representa una tragedia social que impacta las dinámicas familiares, económicas y sociales de los países 1. Por ello, el estudio de los intentos de suicidio y de las muertes por esta causa ha cobrado relevancia, en la medida que se requiere comprender el fenómeno, sus causas y las posturas teóricas que orientan la toma de decisiones y el diseño de estrategias de prevención, atención y mitigación 2.

No obstante, la comprensión del intento de suicidio y del suicidio consumado va mucho más allá de la explicación hegemonizada durante más de dos décadas, proveniente de la epidemiologia clásica, que justifica la ocurrencia de los intentos de suicidio y el suicidio desde la interacción de múltiples factores de riesgo, en los que se fundamentan diversos modelos explicativos con enfoque biomédico-céntrico, que postulan a la conducta suicida como una consecuencia de la psicopatología individual.

Otras miradas del fenómeno han afirmado que los suicidios no siempre ocurren en el contexto de una psicopatología, sino en momentos de tensiones graves, que sobrepasan las habilidades de afrontamiento y resolutividad de las personas, e incluso por ausencia de soporte social 3. Según Ortiz 3, la desigualdad social entre los países de América Latina y en el interior de sus territorios influye directamente en la distribución de todos los fenómenos de salud, debido a que la riqueza suele estar concentrada en una poca proporción de los habitantes de un país, lo que determina la forma en que los colectivos sociales enferman y mueren.

Los países ricos tampoco están exentos del problema, en estos el suicidio puede estar asociado a factores como el aburrimiento que produce la ociosidad y la sobresaturación de deseos y metas alcanzadas, la urbanización, el espíritu de competición y el incremento del individualismo 4.

Además, en sociedades no occidentales con alta riqueza como Japón, el suicidio tiene una clara diferenciación, puesto que muchos ocurren en el contexto de rituales con clara connotación cultural (seppuku o harakiri), como escapar a la humillación del enemigo en caso de derrota, seguir al maestro hacia la muerte o expiar una culpa 4.

Lo anterior conlleva la necesidad de profundizar en la comprensión de estas variables sociales con respecto a el fenómeno del suicidio. Por ello, este ensayo desarrolla una crítica a la postura teórica hegemónica biomédica y resalta las bondades que tendría el estudio del fenómeno desde una perspectiva social, específicamente a partir del modelo de los determinantes sociales de la salud de la Organización Mundial de la Salud (OMS), sin desmeritar los aportes teóricos y empíricos del enfoque biomédico, pero sí destacando sus debilidades, centradas unívocamente en lo psicopatológico, y resaltando las fortalezas del enfoque social, que sin dejar de lado los factores individuales, ofrece una visión más integral en la comprensión de la conducta suicida.

## Explicaciones biomédicas del intento de suicidio y del suicidio consumado

**Figura 1:**
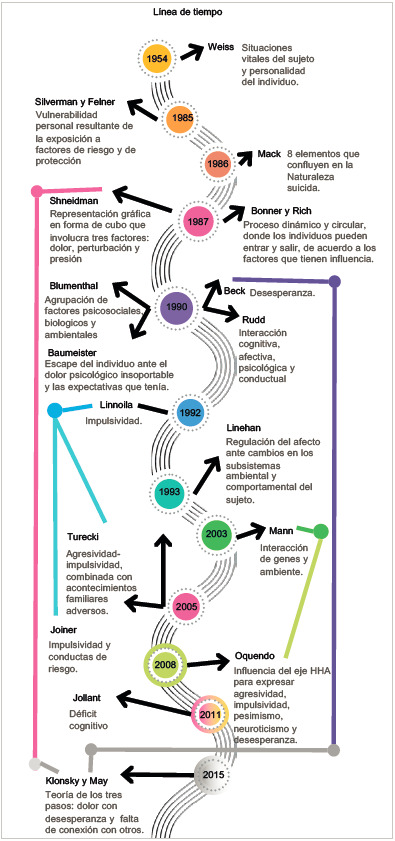
Línea de tiempo y relación de modelos biomédicos para la explicación de la conducta suicida

Desde la década de 1950 ha emergido un cúmulo de modelos biomédicos que han buscado fortalecer dicho enfoque. Estos se organizan, básicamente, desde dos tipos de factores: los netamente psicológicos y los neuro-bioquímico-biológicos (Figura 1).

El común denominador de los modelos radica en considerar que el intento de suicidio y el suicidio se producen por una variabilidad de factores individuales que tienen su base en la dimensión biológica del individuo, por lo que centran su interés en el análisis del funcionamiento neurológico, bioquímico, fisiológico y genético.

Si bien hay modelos biomédicos que postulan otro tipo de factores, que tienen que ver más con la dimensión psicológico-cognitiva del sujeto, como la desesperanza, la impulsividad o la agresividad 5,6, la explicación para la experimentación de estos procesos en el estado mental del individuo sigue siendo naturalista y reduccionista. Así, confiere responsabilidad del desenlace fatal exclusivamente al sujeto, ya sea porque un trastorno mental lo condicionó al evento, porque los factores psicológicos y cognitivos de este superaron la capacidad individual de afrontar las adversidades, o porque confluyeron ambas condiciones que llevaron a la persona al desequilibrio mental y por ende al suicidio.

Pese a los esfuerzos de este enfoque por demostrar la relación causal del suicidio desde el naturalismo, buscando marcadores cerebrales, bioquímicos o genéticos fiables y específicos de la conducta suicida, se destaca su debilidad ontológica. Asume dicha conducta como un síntoma más de una enfermedad o de un desequilibrio biológico-neuroquímico, y a través de asociaciones o correlaciones para inferir causalidad 7,8, cuya investigación impide confirmar del todo esta relación causal.

Además, con base en la epidemiologia clásica, son numerosos los estudios que afirman que la conducta suicida responde a una multicausalidad 9-11; que es un fenómeno complejo que requiere una visión más amplia para su comprensión, y para que ocurra deben interactuar una serie de factores de riesgo que no solo involucran la dimensión biológica del sujeto, sino también la psicológica, la social e incluso la espiritual. Aunque la supremacía se concentra en lo psicopatológico (trastornos mentales), los otros factores actuarían como detonantes o predisponentes.

## ¿Es la psicopatología responsable de todos los intentos de suicidio y del suicidio consumado?

La relación causal entre la conducta suicida y los trastornos mentales, a pesar de los numerosos aportes de estudios teóricos y empíricos que la defienden, sigue siendo difícil de probar.

Un metaanálisis de 65 estudios, con 27 340 individuos con trastorno depresivo mayor, encontró que la prevalencia del intento suicida fue del 31%, es decir, por cada 100 pacientes con trastorno depresivo mayor, 31 a lo largo de su vida intentaron el suicidio 12. Para el caso del trastorno bipolar, un metaanálisis de 79 estudios, con 33 719 sujetos, reportó que la prevalencia del intento de suicidio fue del 33,9% y se asoció positivamente con el género femenino, trastorno bipolar I, nivel de ingresos y región geográfica 13. En el trastorno límite de la personalidad (TLP), caracterizado fuertemente por impulsividad e inestabilidad emocional, se considera que la relación entre la conducta suicida y el TLP es del 10% 14.

Todo este despliegue empírico en torno a la asociación entre el intento de suicidio y suicidio y los trastornos mentales, redunda en una postura que busca validar la conducta suicida ligada exclusivamente al sujeto. De ahí que el positivismo biomédico hegemónico, aunque enuncie multifactorialidad en la ocurrencia de los suicidios y los trastornos mentales, confiere supremacía a la sintomatología individual, como es el caso de la patología psiquiátrica.

Los estudios actuales, aportados en párrafos anteriores, no han podido probar directamente dicha casualidad, ni que el "el 90% de los individuos que intentan suicidarse o mueren por esta causa tienen un trastorno mental", como lo sostuvo la OMS en el 2014, debido a que muchos de dichos estudios informan cifras inferiores al 50%. Aguirre 9 informó una relación del 27% (RP 2,79, ¡095% 1,53-5,1, p=0,01) con consumo de sustancias psicoactivas en adolescentes colombianos; Álvarez 15 reportó 18,9% (p=0,00) con depresión y 27,0 % (p=0,02) con dependencia del alcohol; y Cañón 16 informó asociación de 42,3% entre intentos de suicidio y ansiedad (RP 8,94, p=0,000) en jóvenes colombianos.

Incluso Liang 17, que examinó la asociación entre mortalidad por suicidio y trastornos mentales en el periodo 2000-2014 en población china, concluyó que no hubo correlación significativa entre estas dos variables en todo el periodo de estudio (r=0,447, p=0,10). También Rockett 18 analizó la frecuencia de los antecedentes psiquiátricos en una cohorte de suicidios consumados de EE.UU entre el 2011 y el 2013, y reportó que de 36 190 casos de muerte por suicidio, el 28,4% tenía antecedentes de depresión, el 1,7% de ansiedad y el 3,8% de trastorno afectivo bipolar.

Estos datos reflejan la variabilidad de las estimaciones empíricas y demuestran la poca confiabilidad de respaldar la teoría de que todos o la mayoría de los intentos de suicidio y el suicidio consumado son producto del antecedente personal de una psicopatología. De allí que en la actualidad no se podría afirmar y seguir repitiendo de continuo que la enfermedad mental, cualquiera que sea, es la causa de la conducta suicida, pues los aportes teóricos y empíricos de los últimos 30 años son variables, y aun contradictorios, como se ha enunciado en párrafos anteriores.

## Una apuesta explicativa diferente

Si bien, históricamente, el enfoque biomédico ha prevalecido para explicar la génesis del fenómeno del suicidio en el mundo occidental, sus propias limitaciones han llevado a que en los últimos años se generen explicaciones alternativas como las psicoanalíticas o las sociológicas 19, la determinación social y las fenomenológicas 20, que buscan llenar los vacíos de conocimiento de este enfoque.

El primero en reconocer el papel de lo social en la conducta suicida fue Durkheim en 1897, quien afirmó que las tasas de suicidio no son el resultado único de factores intrínsecos en el sujeto que consuma el acto y, más específicamente, no son unívocamente los trastornos mentales la causa de las muertes por suicidio 19.

Aunque estos aportes desde lo social han sido criticados por Alvira 21, las fortalezas de los aportes de Durkheim y de todos los estudios enunciados que siguen sus postulados se sustentan en la búsqueda más allá de los datos superficiales que vinculan los trastornos mentales con el suicidio, y abren la posibilidad de una explicación social, donde son las condiciones sociales las que determinan la conducta suicida.

La epidemiologia social anglosajona promueve la comprensión de la salud desde las realidades histórico-sociales, pues son las condiciones de vida las que determinan cómo los sujetos transitan por la enfermedad y la muerte 22.

Estos aportes han demostrado que todos los problemas de salud que afectan al ser humano tienen una base social; postulan que así como hay unos determinantes biológicos y comportamientos individuales, en el caso de la conducta suicida, la exposición a trastornos mentales y otras vulnerabilidades internas, también están los sociales, los cuales no dependen de la voluntad individual, sino que se configuran con las relaciones y la interacción humana, y en esa medida se constituyen inequidades en salud debido a esos determinantes sociales 22.

En este sentido, siguiendo los postulados de Durkheim 19 y aquellos de la epidemiología social anglosajona, la conducta suicida requiere también una comprensión social que permita identificar el papel de las desigualdades en la génesis o distribución poblacional de los intentos de suicidio y las muertes por suicidio, entendiendo que este fenómeno requiere que se profundice en

la comprensión del entramado de lazos sociales aunados a las características individuales que determinan el desenlace de los individuos.

El modelo de los determinantes sociales de la salud establece una jerarquía y diferencia dos grandes determinantes subsumidos uno en el otro. En primer lugar, los determinantes estructurales, debido a la posición social, los contextos sociales, políticos, económicos y culturales 23. En segundo lugar, los determinantes intermedios, asociados con las exposiciones y vulnerabilidades diferenciales individuales 23.

Si bien entre los determinantes intermedios del modelo se involucran los trastornos mentales como factor de riesgo individual, cabe destacar que dicho factor no se entiende como un riesgo unívocamente individualista y direccional con la conducta suicida. Más bien, es un determinante, en relación con los otros factores del modelo que se conjugan para generar la conducta suicida, y por ende ofrece una explicación más amplia del fenómeno, no centrada en la psicopatología, sino en aquellas condiciones sociales que podrían interactuar o no con la psicopatología del sujeto cuando esté presente.

Pese a la gran oportunidad que brinda este modelo social de la OMS para explicar el problema del intento de suicidio y la muerte por suicidio, el abordaje investigativo en torno al tema ha sido escaso. Las pocas investigaciones existentes se han centrado en aspectos individuales del sujeto, sin realmente comprender ni explicar la relación entre dichos determinantes individuales y las condiciones sociales del sujeto que realmente generan las inequidades en salud 2,24-30.

Dichas investigaciones se caracterizan por ser de tipo ecológico (en seis de las ocho investigaciones). Ninguno de los estudios analizó de manera integral todos o por lo menos la mayoría de los determinantes; seis se concentraron en describir los estructurales, sobre todo lo referente al índice de pobreza, la edad, el sexo y el nivel educativo; dos investigaciones relacionaron algunos determinantes intermedios como violencia y familia. La mayoría analizó el fenómeno del suicidio consumado (seis investigaciones) (Tabla 1).

**Tabla 1:** Estudios latinoamericanos sobre determinantes sociales en salud y conducta suicida

Autor, año, país	Tipo de estudio y de conducta. Población	Determinantes sociales en salud	Resultados
Campo A, Herazo E. 2014. Colombia 24	Estudio ecológico. Suicidio consumado. No informa.	Estructurales: índice de pobreza (coeficiente Gini)	La correlación entre pobreza (r=-0,401; p=0,052) y coeficiente de Gini y (r=-0,086; p=0,689) y la tasa de suicidio no fue significativa.
Ordoñez I, 2021, Colombia 2	Estudio ecológico. Suicidio consumado. Adultos mayores.	Estructurales: edad, sexo, zona de residencia, régimen de afiliación al sgsss, nivel educativo, estado civil y pertenencia étnica	El riesgo de suicidio es 8,14 veces mayor en hombres que en mujeres. A medida que aumenta la edad (mayor a 80) disminuye el riesgo de suicidio. Separados y divorciados. Residentes de zonas urbanas. No asegurados (OR=7,52) y sin identificación de régimen de afiliación (OR=32,01), educación superior.
Goñy W y Rivas J, 2018, México 25	Estudio analítico transversal. Ideación suicida. Estudiantes de secundaria.	Intermedios: salud, escuela, familia, vivienda.	Relación lineal moderada y directamente proporcional entre los dss-intermediarios y el nivel de riesgo is (rs=,808, p=,000). 7% se ubicó en alto riesgo de is, 39% moderado y 54% bajo, la media mayor en relación al sexo fue en mujeres que en hombres (15,17/12,52). El porcentaje de dss-intermediarios por dimensión, ubico a la familia (42%), vivienda (35%), escuela (39%), salud (51%) como coadyuvantes en el riesgo is.
Salcedo F, 2019. Colombia 26	Estudio analítico transversal. Jóvenes entre 10 y 24 años. Intento de suicidio.	Estructurales: edad, educación, situación laboral. Intermedios: estado civil, convivencia con los padres, maltrato verbal y físico, educación del padre y de la madre, víctima de conflicto (desplazado), mala relación con padres, pensamientos suicidas, consumo de alcohol, consumo de drogas y conductas irritables	Los jóvenes con pensamientos suicidas (U=0,063, p=0,000), aquellos que han sido víctimas de abuso físico (U=0,016, p=0,004), y los que tienes padres con bajos niveles de escolaridad (U=0,014, p=0,004) tienen una mayor probabilidad de atentar contra sus vidas.
Campo A, 2015. Colombia 27	Ecológico. Suicidio consumado. No informa.	Estructurales: índice de pobreza (coeficiente Gini)	La correlación entre desigualdad y tasa de suicidio fue positiva y estadísticamente significativa (r=0,70; p<0,001).
Manríquez 2015. México 28	Ecológico. Suicidio consumado. No informa.	Intermedios: horas de trabajo semanales, acceso a las instituciones de salud. Estructurales: tasa de desocupación, población ocupada, ingreso, gasto en prevención y estancamiento del pib, sexo, edad	El desempleo, el ingreso y la carga laboral obtuvieron coeficientes positivos y estadísticamente significativos, al igual que el no acceso a las instituciones de salud. El incremento en el porcentaje de ocupados aumenta la tasa de suicidio en un 9,0%. El efecto es más marcado en los hombres, con un 11,2%, mientras que en las mujeres no resulta significativo.
Borges D, 2015 Brasil 29	Ecológico. Suicidio consumado. Todas las edades.	Estructurales: índice de Gini, ingreso per cápita, educación, tasa de urbanización, promedio de residentes por hogar, divorciados y filiación religiosa	La desigualdad de ingresos, evangélicos y personas que no terminaron los estudios básicos se asoció positivamente con las tasas de suicidio. El ingreso per cápita, la tasa de urbanización, el número promedio de residentes por hogar y el porcentaje de pentecostales se asoció negativamente con el suicidio.
Dantas A, Brasil 2018 30	Ecológico de múltiples grupos. Suicidio consumado. Toda la población.	Estructurales: indicadores socioeconómicos (índice de desarrollo humano municipal (idh-m); relación de ingresos entre el 10% más rico y el 40% más pobre; índice de Gini; tasa de desempleo entre los > 18 años; porcentaje de población que vive en hogares con densidad)	El Medio Oeste y el Sur tenían las tasas medias de mortalidad por suicidio más altas. De los indicadores socioeconómicos analizados, las peores condiciones se concentraron en el Norte y Nordeste, que se caracterizan por baja esperanza de vida, desigualdades de ingresos, baja educación y bajos ingresos. Las regiones más desarrolladas del país, Sur y Sudeste, se diferenciaban considerablemente de las más pobres.

### Consideraciones finales

No se puede desconocer que existe evidencia empírica y teórica que respalda la relación entre la psicopatología y los intentos y las muertes por suicidio. Se sabe que un número de casos puede estar directamente asociado a cualquier psicopatología, sin embargo, la evidencia es más débil de lo que se ha popularizado, debido a las propias contradicciones del enfoque biomédico y a los datos que evidencian cifras inferiores a las informadas por instituciones con alto poder de autoridad como la OMS.

De otra parte, la evidencia que respalda esta relación causal, proviene de estudios observacionales analíticos transversales 9-11,15,16, la mayoría con limitaciones metodológicas importantes que afectan la validez interna de los resultados. Una de ellas es la selección de los participantes mediante muestreo no probabilístico, con frecuencia, por conveniencia. Este tipo de muestreo suele ser utilizado por los investigadores cuando existe un tiempo limitado para llevar a cabo la investigación o cuando se presentan barreras presupuestarias para la ejecución, sin embargo, este muestreo hace susceptible a los resultados de baja validez interna y externa y confiere debilidad a las asociaciones, puesto que no asegura la representación total de la población y puede carecer de objetividad 31.

Además, estas investigaciones con frecuencia incurren en sesgos metodológicos importantes que les restan confiabilidad. Un ejercicio de valoración de riesgo de sesgo de acuerdo a los dominios para estudios observacionales de la colaboración Cochrane (ROBINS-E) 32, realizado por esta autora a 15 investigaciones que asocian el riesgo suicida con la presencia de depresión, trastornos de la conducta alimentaria, consumo de tabaco, alcohol y drogas ilícitas, demostró que 11 de 15 de estudios tienen un alto riesgo de sesgo, debido a que no se hace un esfuerzo por controlar los factores de confusión que puedan incidir en los resultados y en estas conclusiones. Cabe resaltar que estos estudios incurrieron en sesgos referentes al dominio (a): debido a factores de confusión; al dominio (b): riesgo de sesgo derivado de la medición de la exposición; y al dominio (c): riesgo de sesgo en la selección de participantes en el estudio o en el análisis. Además, seis de quince no pasaron de la valoración preliminar 32 (Tabla 2).

**Tabla 2:** Resumen de valoración de riesgo de sesgos de estudios observacionales robins-e

Estudio	Variables	Valoración preliminar	Dominios							Riesgo de sesgo
			1	2	3	4	5	6	7	
Santis R, 2016 33	Consumo de cocaína	SI	SÍ	NO	SÍ	SÍ	SÍ	SÍ	NO	Alto
Kelleher I, 2017 34	Trastornos psicóticos	NO	-----	-----	-----	-----	-----	-----	-----	Alto
Rodríguez M, 2013 35	Trastorno de la conducta alimentaria	SÍ	SÍ	NO	NA	NA	SÍ	SÍ	NO	Alto
Franko D, 2013 36	Trastorno de la conducta alimentaria	SÍ	SÍ	NO	NA	NA	SÍ	SÍ	NO	Alto
Aguirre D, 2013 9	Depresión, consumo de alcohol y drogas, trastorno de la conducta alimentaria	NO	-----	-----	-----	-----	-----	-----	-----	Alto
Álvarez J, 2012 15	Consumo de sustancias psicoactivas	NO	-----	-----	-----	-----	-----	-----	-----	Alto
Bimala S, 2015 10	Consumo de alcohol y sustancias ilícitas	SÍ	NO	NO	NA	NA	NO	NO	NO	Bajo
Perez I, 2012 37	Depresión	NO								Alto
Silva D, 2017 38	Consumo de tabaco	SÍ	SÍ	NO	NA	NA	SÍ	NO	NO	Alto
Valdivia, 2015 39	Depresión, consumo de tabaco y drogas	SÍ	NO	NO	NA	NA	NO	NO	NO	Bajo
Cañón S, 2017 16	Trastornos mentales	NO	-----	-----	-----	-----	-----	-----	-----	Alto
Álvarez, 2017 40	Depresión y trastorno de conducta	NO	-----	-----	-----	-----	-----	-----	-----	Alto
Cañón S, 2021 41	Depresión	SÍ	SÍ	NO	NA	NA	SÍ	SÍ	NO	Alto
Pérez A, 2020 42	Depresión	NO	-----	-----	-----	-----	-----	-----	-----	Alto
Liang Y, 2018 17	Trastornos mentales	NO	-----	-----	-----	-----	-----	-----	-----	Alto

NOTA: robins-e ofrece inicialmente una valoración preliminar del estudio, consta de cuatro preguntas. Cuando en una de estas preguntas la respuesta es positiva, recomienda no continuar con la valoración de la investigación. Luego, se evalúan siete dominios: (1) debido a factores de confusión, (2) derivado de la medición de la exposición, (3) debido a la selección de participantes en el estudio o en el análisis, (4) debido a las intervenciones posteriores a la exposición, (5) debido a la falta de datos, (6) derivado de la medición del resultado y (7) por selección del resultado informado. Las preguntas de cada dominio se contestan con opciones: SÍ, NO, NI: no informa, NA: no aplica. De acuerdo a la valoración global de los dominios, se clasifica el riesgo de sesgo final del estudio como: muy alto riesgo de sesgo, alto riesgo de sesgo y bajo riesgo de sesgo. A efectos de facilitar la visualización de esta tabla, se resume la valoración de cada dominio, colocando SÍ, cuando hubo sesgo; NO, cuando no estuvo presente el sesgo y NA: cuando por el diseño mismo de la investigación, el dominio no era susceptible de evaluación.

Otro aspecto por destacar es la heterogeneidad de los estudios que establecen estas asociaciones: la mayoría utiliza diferentes cuestionarios de autorreporte, algunos validados, otros construidos por los propios autores de las investigaciones para el análisis de las variables de depresión y consumo de sustancias; solo tres de quince estudios analizados utilizaron la entrevista por personal formado en salud mental para definir trastorno de conducta alimentaria y depresión.

Lo anterior implica que las investigaciones realizadas a partir del enfoque biomédico-psicopatológico incurren en heterogeneidad metodológica, e incluso heterogeneidad estadística, por la variación en la estimación del efecto, lo que hace necesario tomar con precaución los resultados informados para no incurrir en conclusiones sesgadas 43. A su vez, el hecho de que algunas utilicen instrumentos no validados para medir las variables de interés, hace dudar del rigor de la investigación, puesto que las mediciones carecen de validez y confiabilidad 44. En contraste, cuando los instrumentos autoinformados reportan su fuente de validez, se garantiza una adecuada interpretabilidad de las conclusiones extraídas del estudio, así como reconocer sus debilidades.

Por otro lado, es importante destacar la ausencia de investigaciones realizadas con base en el enfoque biomédico que diferencien el efecto de las variables de exposición sobre las variables resultado. En este caso, las variables de exposición son los trastornos mentales y el antecedente de trastorno mental en la familia, que podrían tener efectos diferentes en cada una de las fases de la conducta suicida, como la ideación, el intento y el suicidio consumado. Los estudios con frecuencia analizan la asociación de las variables de exposición, sin discriminar claramente las variables de desenlace. Si se considerara el estudio diferencial de la exposición sobre las diferentes variables resultados, se podría controlar el sesgo de confusión 45.

De otra parte, las investigaciones desde lo social para explicar la conducta suicida han sido escasas, sin embargo, en este ensayo se mostró que, desde Durkheim, durante más de cien años se han hecho contribuciones que sugieren la relación de la conducta suicida con los factores sociales, que destacan que el suicidio es más bien una consecuencia de las características sociales actuales, inmersas en la desigualdad. Pese a estos esfuerzos, pareciera que estos aportes se hubieran invisibilizado en la comunidad científica, para dar supremacía al enfoque biomédico.

No obstante, tal hegemonía ha permitido avanzar en el diseño de intervenciones de prevención y atención a los sujetos con trastornos mentales. Se espera que si estos son diagnosticados oportunamente, reciban un tratamiento continuo y de calidad, se reduzca el riesgo de que el evento suicida ocurra. A su vez, del trabajo que se debe hacer con la familia para que brinde soporte al tratamiento del paciente, la reducción del estigma a la salud mental, la reducción de barreras de acceso para la atención en salud mental, ente otras 46.

Lo anterior deja de lado aquellos intentos y suicidios que no tuvieron ninguna relación con la psicopatología, aquí el abordaje de prevención y atención debe ser diferente. Según Rose, el futuro biomédico que respalda la psiquiatría contemporánea tiene la necesidad imperativa de abordar los determinantes sociales y políticos de los trastornos mentales 47. En este sentido, la investigación de la conducta suicida debe avanzar a profundizar cómo los procesos sociales, en este caso los determinantes sociales de salud, interactúan en la génesis de la conducta suicida o condicionan la ocurrencia del fenómeno desde el punto de vista de la desigualdad social, donde el trastorno mental, si está presente, no es un factor aislado, sino que interactúa con otros para llevar al evento ♠
